# Apply the Hirano or Japanese criteria when diagnosing MELAS

**DOI:** 10.1016/j.amsu.2022.104965

**Published:** 2022-11-17

**Authors:** Josef Finsterer, Sounira Mehri

**Affiliations:** a Neurology & Neurophysiology Center, Vienna, Austria; b Biochemistry Laboratory, LR12ES05 “Nutrition-Functional Foods and Vascular Health”, Faculty of Medicine, Monastir, Tunisia

**Keywords:** MELAS, mtDNA, Stroke-like episode, Myopathy, Lactic acidosis, EEG, electroencephalography, FSGS, focal segmental glomerulosclerosis, MELAS, mitochondrial encephalopathy, lactic acidosis, and stroke-like episode, MID, mitochondrial disorder, RRF, ragged-red fibers, SDH, succinate dehydrogenase, SLE, stroke-like episode, SLL, stroke-like lesion, SNHL, sensorineural hearing loss, TIA, transitory ischemic attack, WML, white matter lesion, COC, cytochrome-C oxidase

Letter to the Editor

We read with interest the article by Alsultan et al. reporting on a 33 years-old male with mitochondrial encephalopathy, lactic acidosis, and stroke-like episode (MELAS) syndrome with an unusual phenotype [1]. Uveitis, pigmentary retinopathy, and focal segmental glomerulosclerosis (FSGS) have been identified as unusual phenotypic features [1]. The study is attractive but raises concerns that should be discussed.

The main limitation of the study is that the diagnosis of MELAS or mitochondrial disorder (MID) was not genetically confirmed [1]. The diagnosis of a MID without proof of a corresponding genetic defect is possible in individual cases, but only the proof of a suitable genetic defect eliminates diagnostic uncertainties. Even using the Hirano criteria [2] to diagnose MELAS (stroke-like episode (SLE) < age 40, seizures or dementia, lactic acidosis or ragged-red fibers, normal early development, recurrent headache, recurrent vomiting), the index patient had no MELAS. The index patient also had no MELAS according to the Japanese criteria [3], since no genetic defect was found.

Another limitation is that the patient has apparently never from a SLE [1]. SLEs are the phenotypic hallmark of MELAS [4]. SLEs are the clinical correlate of so-called stroke-like lesions (SLLs) on cerebral MRI [4]. Since SLLs can be triggered by seizures, we should know if he patient has ever had a seizure and if the electroencephalography (EEG) after the transitory ischemic attack (TIA) showed epileptiform discharges. Particularly, we should know if the TIA was in fact a focal seizure followed by Todd paralysis.

We disagree with the notion that the patient's periventricular white matter lesions (WMLs) were due to atherosclerosis [1]. WMLs are a common abnormality in MID patients and are commonly found even in patients with no cardiovascular risk profile.

We also disagree that the patient had RRFs. RRFs are diagnosed using Gomori trichrome staining rather than Hematoxylin-Eosin stain. Therefore, it remains uncertain whether the patient actually had RRFs. We should be informed whether an SDH straining or COX stain was additionally applied. We should also know if muscle biopsy has been subjected to a biochemical study to assess the activity of respiratory chain complexes.

We disagree with the notion that FSGS is a rare phenotypic feature of MELAS [1]. Several cases in unrelated MELAS families with FSGS have been reported [5].

There is a discrepancy between the neurologic exam at age 32 with no apparent hearing loss and the diagnosis of white matter lesions (SNHL) [1]. We should be informed about the results of the audiogram of the index patient.

At age 32, the index patient suffered a TIA with right hemiplegia and dysarthria treated with aspirin, rivaroxaban, and atorvastatin [1]. It is crucial to know what type of cerebral imaging was applied to diagnose the TIA, whether cerebral imaging was normal as it should be in a TIA, and how a SLL lesion was ruled out. The indication for the simultaneous administration of aspirin and rivaroxaban, should be explained and whether there was atrial fibrillation. Additionally, we should be informed about the cardiovascular risk profile of the index patient in addition to hyperlipidemia, and whether he was a smoker.

Why didn't the patient go to the doctor earlier? It is inexplicable why someone with urinary retention did not visit the urology ward sooner. The patient had edema for about 1 year [1]. Were any investigations carried out before the admission described in the article?

What caused hypertonia of the left upper limb? Furthermore, was hypertonia characterised as rigor or spasticity? Basal ganglia calcifications hardly explain unilateral hypertonia since they occurred bilaterally.

Since uveitis has not been previously reported as a manifestation of MELAS, it is quite unlikely that the uveitis in the index patient was causally related to MELAS. We should know which differential diagnoses of uveitis have been ruled out. At the age of 32 the patient was diagnosed with “undefined vasculitis” [1]. We should know in which region vasculitis occurred or if it was generalised. It is also important to know how the vasculitis was diagnosed, particularly whether vascular or tissue biopsies were taken that confirmed the diagnosis. Uveitis and vasculitis are the only two features that have not been described in MELAS patients (Table 1). For this reason, they may not be related to MELAS or may be misdiagnosed.

**Table 1:** Phenotypic manifestations of MELAS in the index patient and previously described.

	Index patient	Previously described
Short stature	yes	yes
Migraine	yes	yes
Basal ganglia calcification	yes	yes
Tetraspasticity	yes	yes
Extrapyramidal syndrome	yes	yes
Cataract	yes	yes
Pigmentary retinopathy	yes	yes
Uveitis	yes	no
Hearing loss	yes	yes
Vasculitis	yes	no
Renal insufficiency	yes	yes
Myopathy (ptosis, RRF)	yes	yes

RRF: ragged-red fibers.

Overall, the interesting study has limitations that challenge the results and their interpretation. Clarifying these weaknesses would strengthen the conclusions and could improve the study. Diagnosing MELAS requires not only the presence of an appropriate phenotype but also documentation of a causal genetic defect.

## Funding sources

No funding was received.

## Ethics approval

Was in accordance with ethical guidelines. The study was approved by the institutional review board.

## Consent to participate

Was obtained from the patient.

## Consent for publication

Was obtained from the patient.

## Availability of data

All data are available from the corresponding author.

## Code availability

Not applicable.

## Author contribution

xx: design, literature search, discussion, first draft, critical comments, final approval, MS: literature search, discussion, critical comments, final approval, Provenance and peer review: not commissioned, externally peer reviewed.

## Registration of research studies

1. Name of the registry:
2. Unique Identifying number or registration ID:
3. Hyperlink to your specific registration (must be publicly accessible and will be checked):

## Guarantor

Finsterer Jz.

## Declaration of competing interest

None.
